# Barriers to healthcare utilization among married women in Afghanistan: the role of asset ownership and women’s autonomy

**DOI:** 10.1186/s12889-024-18091-y

**Published:** 2024-02-26

**Authors:** Manzoor Ahmad Malik, Ratnesh Sinha, Angelin Priya, Mohammad Hifz Ur Rahman

**Affiliations:** 1 Center for Applied Health Economics, Menzies Health Institute, Griffith University, Queensland, Australia; 2 Department of Community Medicine, Manipal Tata Medical College, Manipal Academy of Higher Education, Manipal, India

**Keywords:** Maternal health, Healthcare, Healthcare utilization, Health systems, Women’s health, Afghanistan

## Abstract

Women face multiple socio-economic, cultural, contextual, and perceived barriers in health service utilization. Moreover, poor autonomy and financial constraints act as crucial factors to their healthcare accessibility. Therefore, the objective of the present study is to study the association between health care utilization barriers and women empowerment, including asset ownership among currently married women in Afghanistan. Data of 28,661 currently married women from Afghanistan demographic health survey (2015) was used to carry out this study. Barriers to access healthcare were computed based on problems related to permission, money, distance, and companionship, whereas women empowerment and asset ownership were computed as potential covariates along with other socio-economic risk factors. Bivariate and logistic analysis was carried out to study the association and odds of explanatory variables. Our results confirm the significant and strong association between the barriers to access healthcare and various explanatory variables. Women having any decision-making autonomy are less likely to face any odds [(AOR = 0.56, *p* < 0.001), CI: 0.51–0.61] among the currently married women than those who don’t have any decision-making authority. Similarly, women who justify their beating for some specific reasons face the greater difficulty of accessing health care [(AOR = 1.76, *p* < 0.001), CI: 1.61–1.93]. In terms of asset ownership, women having any asset ownership (land or household) are less likely to face any barriers in health services utilization given the lower odds [(AOR = 0.91, *p* < 0.001), CI: 0.90–0.98]. Accessing maternal health is a crucial policy challenge in Afghanistan. A substantial proportion of women face barriers related to approval, money, distance, and companionship while accessing the health services utilization in Afghanistan. Similarly, women empowerment and asset ownership are significantly associated with health service accessibility. This paper therefore suggests for some policy interventions to strengthen the healthcare needs of women and ensure healthcare accessibility by scaling down these potential barriers like poor autonomy, asset ownership and domestic violence.

**Supplementary Information:**

The online version contains supplementary material available at 10.1186/s12889-024-18091-y.

## Introduction

 Maternal health remains a pressing global concern despite substantial advancements in health systems during the 21st century [1]. Regions such as South Asia and Sub-Saharan Africa continue to grapple with profound challenges, marked by significant disparities in maternal health outcomes [2, 3]. This inequality is attributed to various socio-economic and cultural factors encompassing inadequate pregnancy methods, a lack of pre and post-natal care, healthcare costs, economic constraints, and limited women’s autonomy [4–6]. Additionally, various barriers impede access to healthcare services, contributing to health disparities and increased mortality risk among reproductive women [7–9].

Afghanistan is one of the most war-torn countries globally, characterized by a fragile public health system and constrained healthcare availability and accessibility [10, 11]. Women in Afghanistan face heightened vulnerability, experiencing a greater likelihood of healthcare vulnerability and disease burden [12–14]. Basic health services and information related to family planning and reproductive health are often inaccessible to women in Afghanistan, with a substantial proportion of births going unattended by healthcare professionals [15, 16]. Moreover, a significant portion of currently married women lacks essential pre and post-natal care [17]. A large proportion of births are still unattended by health care professionals, whereas pre and post-natal care is also lacked by a large proportion of currently married women [18]. Existing studies highlight pronounced inequality in the utilization, availability, and accessibility of healthcare services across socio-economic groups in Afghanistan [2, 19]. Despite efforts to address maternal health, persistent challenges prevail, such as low utilization of antenatal care and a concerning lack of post-natal care among married women in Afghanistan [20]. Women’s also face multiple other challenges to access these health services involving money, transportation and cultural barriers, including poverty, awareness and cultural rigidity, which make them susceptible to access these basic health services [21, 22]. The fragile health system in Afghanistan, heavily reliant on foreign donors, exacerbates challenges in healthcare utilization, both in terms of availability and accessibility [22]. While previous studies shed light on the broader challenges of maternal healthcare, ante natal care and other predictors of healthcare utilization in Afghanistan [18, 23, 24]. There is a conspicuous gap in research specifically examining the barriers to accessing these healthcare services. Considering this, this study seeks to fill this gap by conducting a comprehensive analysis of the barriers to health service utilization among currently married mothers in Afghanistan. We aim to explore associated risk factors, including women’s empowerment and asset ownership, factors that have not been extensively studied in this context. By addressing these gaps in the literature, our research aspires to contribute valuable insights that can inform targeted interventions and policies to improve maternal health outcomes in Afghanistan. Furthermore, this research can serve as a foundation for crafting fresh initiatives and underscore the importance of conducting sociodemographic surveys in Afghanistan.

### Literature review

Barriers to access healthcare services are a crucial concern in public policy research. Studies on healthcare service equality and excess have primarily focused on coverage indicators, neglecting analysis of individuals who encounter challenges in accessing healthcare services despite their availability [25]. Various perceived barriers contribute to the underutilization of healthcare services among reproductive women. Studies have explored factors impeding progress in healthcare service accessibility, including socio-economic vulnerability, affordability, and the availability of health services in developing countries, such as Afghanistan [26]. Similarly, a range of barriers from cultural to contextual barriers hinders free and equitable access to healthcare services, including issues related to women’s empowerment [27, 28]. Other obstacles in seeking healthcare, such as time constraints, unwillingness due to cultural norms, and associated costs, also impact women’s health [29]. Furthermore, additional studies have identified critical factors like women’s empowerment and asset ownership due to their close association with the utilization of maternal health services [27–29].

Previous studies have documented the close association between women’s health and their socio-economic settings [30, 31]. Women with greater autonomy and higher status are likely to have increased freedom to access healthcare [32]. Similarly access to education, employment, and resource ownership is crucial for women’s well-being and overall sustainable development [33]. Moreover, greater access to healthcare is directly dependent on women’s empowerment and socio-economic well-being [34]. Research indicates that women with a more significant role in decision-making are likely to have the freedom to choose healthcare services available to them [35]. Empowered women are more likely to have better access to healthcare services, facing fewer financial and companionship constraints when seeking healthcare services [36].

Afghanistan faces challenges in both socio-economic and healthcare services on a large scale. Women’s empowerment is a highly debated issue in Afghanistan, particularly considering the impact of conflict over the years [19]. There is limited knowledge about women’s empowerment and their significant role in the Afghanistan given the fragile environment. Similarly, little exploration has been undertaken to study the connection between women’s status and healthcare service utilization. Although health indicators have significantly improved over the past decade in Afghanistan, the country’s health system remains vulnerable [37]. Afghanistan is still far from achieving the sustainable development goals given the high rates of child and maternal mortality [38]. Similarly, the utilization of child and maternal health services is suboptimal within the country [39]. Afghanistan exhibits the highest maternal health risks in South Asia, with women facing significant challenges in accessing healthcare [18]. Therefore, this study aims to examine the association between women’s empowerment, asset ownership, and the challenges women encounter in accessing healthcare services in Afghanistan.

## Data and methods

This study utilized the Afghanistan demographic health survey AFDHS- 2015 data conducted by the Central Statistics Organization and Ministry of Public Health Afghanistan. The detailed information about the survey, sampling design and available indicators is provided at https://dhsprogram.com [40, 41]. AFDHS is the first standard demographic and health survey conducted in Afghanistan collecting information on a broad range of issues on demographic and health indicators such as family planning, maternal and child health, the nutritional status of women and children, and knowledge and attitudes about HIV/AIDS and domestic violence and so on [41]. Although this survey was conducted in 2015, but it is the latest available comprehensive survey on health and wellbeing indicators in country. Thus, in this scenario, the study findings can be used as baseline research to develop new proposals as well as to highlight the need for conducting sociodemographic surveys to have access to updated data from Afghanistan.

### Outcome variable

Access to health care was the primary dependent variable in our study. The AFDHS recorded a set of information on barriers to access healthcare among currently married women. These include the following four variables 1; permission to go to the doctor 2; getting money for receiving treatment, 3; distance from health facility and 4; not wanting to go alone. All four variables were binary. Therefore, we combined them to create a binary variable with (0 = no barriers at all, 1 = faced any barrier). Since the main aim of the study was to estimate the probability of one outcome relative to the other rather than making a comparison of probabilities across categories, so we employed the logistic model. The detailed information about dependent variables and question used for collected in this survey are provided in the Additional file 1.

### Exposure variables

Two key exposure variables were considered in this study based on the available information in the study. First, we computed the women’s empowerment based on two dimensions involving decision making and reasons for justifying the beating as used by earlier studies [28]. While women empowerment is a multidimensional measure involving measures related to various key factors such as economic participation, work opportunity, political empowerment, educational attainment and health and wellbeing [42]. We included only two of the critical variables, decision making and reasons for justifying beating as a proxy for women empowerment in our study. These variables provide better picture of women’s empowerment and their self-dependency than the factors like level of education and labor force participation [40–42]. Similarly, many studies have also included these measures in their respective studies, since both they assess freedom and autonomy in decision making of women [43, 44]. The other measure included in the study was asset ownership, which was computed from house and land ownership in this study. These two ownerships are strongly associated with greater autonomy of women and their better status in society [45, 46]. The detailed information on both variables is given in the Additional file 1.

### Other covariates

The other covariates selected in the study were determined based on the available literature. These included a set of socio-demographic characteristics of the mothers: maternal age, number of living children, level of education for both mother and father, work status, Place of residence, wealth index and other covariates. The detailed account of these variables can be found in the Table T1 Additional file 1.

### Statistical analysis

Bivariate analysis was carried out to study the relationship between variables of interest. We used chi-square test to study the association between our dependent variables (barriers to access health care) with independent variables like women empowerment, and asset ownership and similarly other independent variables which include socio-economic, demographic, and other contextual factors. The Chi-square test was measured at the 5% level of significance (alpha = 0.05). A logistic model was then computed to examine the risk factors associated with any barrier among the currently married women in the present study. The results were reported in adjusted odds ratios (AOR) at 1, 5 and 10% levels of significance respectively adjusted for various socio-economic and associated risk factors. All analysis was carried using Stata 15 in this paper.

## Results

**Fig. 1:**
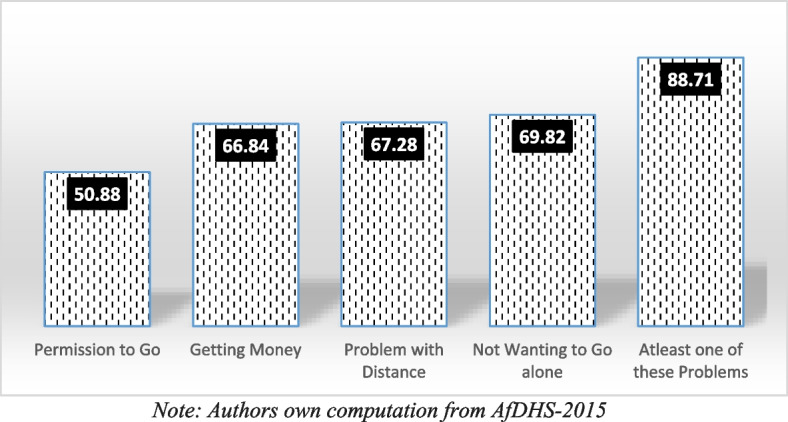
Problems faced by currently married women in accessing health care services in Afghanistan

Figure 1 shows the barriers faced by currently married women in Afghanistan. Of the total sample (28,671) more than 88% currently married women face any problem in utilization of healthcare services in Afghanistan. Around 70% women face any problem to access healthcare due to not being accompanied by anyone. The figure also shows that 67% of women also face problems due to being distant healthcare facility. Similarly financial challenges account for 66%, whereas 50% face any barrier due to lack of permission to go out.

Table 1 shows the barriers to healthcare access among currently married women in Afghanistan, categorized by socio-economic status, women’s empowerment, and ownership of assets. Higher barriers were found among women belonging to marginalized groups. Only 55% of women with higher education faced any barriers compared to 91% of those without any education. Similarly, 87% of women with decision-making power faced barriers compared to 91% without such authority. Only 9% of women who owned land did not faced any problems in accessing healthcare, compared to 12% who did not own any land. The chi-square value at the bottom of the table indicates that all these factors were significantly associated with barriers to healthcare access at a 5% level of significance (*p* < 0.05).

**Table 1:** Barriers in accessing health care services according to socio-demographic characteristics, women empowerment indicators and asset ownership by currently married women in Afghanistan (AfDHS − 2015)

Background Characteristics	Problems in Accessing Healthcare		
	**No Problem (%)**	**Any Problem (%)**	**Sample (N)**
**Age Group**			
15–19	8.80	91.20	1,811
20–29	10.90	89.10	12,339
30–39	12.00	88.10	8,593
40–49	11.30	88.70	5,918
**Place of Residence**			
Urban	22.20	77.90	6,815
Rural	8.00	92.00	21,846
**Number of Living Children**			
0	10.60	89.40	2,931
1–2	12.40	87.60	6,917
3–4	11.00	89.00	7,448
5+	11.00	89.00	11,365
**Mothers Education**			
No Education	8.70	91.30	24,488
Primary Education	18.90	81.00	1,928
Secondary Education	24.90	75.10	1,761
Higher Education	45.00	55.00	484
**Mothers Occupation**			
Not Working	10.70	89.30	25,673
Working	14.60	85.40	2,988
**Husband Education**			
No Education	8.60	91.40	16,132
Primary Education	12.20	87.80	3,826
Secondary Education	13.90	86.20	6,362
Higher Education	23.60	76.40	2,341
**Ethnicity**			
Pashtun	6.60	93.40	12,232
Tajik	14.10	85.90	8,686
Hazara	10.70	89.20	2,658
Uzbek	20.20	79.80	1,978
Turkmen	14.40	85.60	619
Nuristani	2.50	97.50	1,180
Baloch	17.40	82.60	386
Pashai	5.70	94.40	503
Other	11.20	88.80	419
**Wealth Index**			
Poorest	5.70	94.30	5,491
Poorer	8.00	92.00	6,555
Middle	9.20	90.80	6,182
Richer	10.70	89.40	6,104
Richest	23.40	76.60	4,329
**Decision Making**			
No	8.37	91.63	10,802
Yes	12.83	87.17	17,859
**Reason for Wife Beating**			
No	21.21	78.79	5614
Yes	8.90	91.10	23,047
**Owns Land**			
No	12.08	87.92	19,081
Yes	9.01	90.99	9580
**Owns House**			
No	10.39	89.62	14,801
Yes	12.69	87.31	13,860
Total	**11.29**	**88.70**	**28,671**

Authors own computation from AfDHS-2015

Table 2 presents the results for risk factors associated with barriers to accessing health care among women in Afghanistan. AOR stands for adjusted odds ratio which measures the strength of the association between a particular exposure or risk factor and an outcome, while controlling for the effects of other variables that may influence the relationship. The results in the Table 1 shows that women aged 40–49 are more likely to access health care services than the women aged 15–19 [(AOR = 0.53, *p* < 0.001), CI: 0.42–0.67]. Similarly, rural women are more likely to face barriers to access healthcare [(AOR = 2.08, *p* < 0.001), CI: 1.85–2.34] as compared to urban women. Working women [(AOR = 0.82, *p* < 0.001), CI: 0.72–0.94] and women with higher education [(AOR = 0.76, *p* < 0.001), CI: 0.65–0.88] are less likely to face the healthcare excess barriers as compared to women who are not working and are illiterate respectively. Regarding the wealth index, as we move towards better affluent groups, odds of having any barrier significantly decreases. Women belonging to richer households are less likely to face any barrier compared to the women belonging to poorest households [(AOR = 0.48, *p* < 0.001), CI: 0.41–0.57]. While examining our exposure variables our results clearly found a significant association between women’s decision-making freedom and barriers to health care accessibility. Women having any decision-making ability are less likely to face any challenges in accessing health care [(AOR = 0.56, *p* < 0.001), CI: 0.51–0.61] as compared to those who are not independent in their decision making on critical issues. We also found that the women’s who justify their beating for some specific reasons face the greater odds of accessing health care [(AOR = 1.76, *p* < 0.001), CI: 1.61–1.93]. In terms of asset ownership, we also found the significant and negative association with women having any asset ownership of facing barriers in healthcare accessibility [(AOR = 0.91, *p* < 0.001), CI: 0.90–0.98].

**Table 2:** Association between problems in health care access, women’s empowerment and asset ownership adjusted for socio-economic and demographic risk factors (AfDHS − 2015)

	Adjusted Odds Ratio	Confidence Intervals	
		Lower Limit	Upper Limit
**Age Group**			
15–19®	Ref		
20–29	0.84*	0.69	1.02
30–39	0.69***	0.55	0.85
40–49	0.53***	0.42	0.67
**Residence**			
Urban®	Ref		
Rural	2.08***	1.85	2.34
**Number of Living Children**			
No Children®	Ref		
Upto Two Children	1.05	0.90	1.23
Three to Four Children	1.1	0.93	1.30
Five and above	1.22**	1.02	1.45
**Mothers Education**			
Illiterate®	Ref		
Primary	0.64***	0.55	0.74
Secondary	0.47***	0.41	0.54
Higher	0.36***	0.28	0.45
**Mothers Occupation**			
Not Working®	Ref		
Working	0.82***	0.72	0.94
**Husband Education**			
Illiterate®	Ref		
Primary	0.92	0.81	1.04
Secondary	0.87**	0.78	0.96
Higher	0.76***	0.65	0.88
**Wealth Index**			
Poorest®	Ref		
Poorer	0.71***	0.61	0.83
Middle	0.66***	0.57	0.77
Richer	0.70***	0.60	0.82
Richest	0.48***	0.41	0.57
**Decision Making**			
No®	Ref		
Yes	0.56***	0.51	0.61
**Wife beating Justified for any one**			
No®	Ref		
Yes	1.76***	1.61	1.93
**Asset Ownership**			
No®	Ref		
Yes	0.91**	0.90	0.98

Authors own computation from AfDHS-2015, ***®***is reference category of independent variables respectively, Not having any problem in accessing healthcare is the reference category for dependent variable, * indicates significance level: ***significant at 1%, **significant at 5%, *significant at 10%, confidence interval in parentheses

## Discussion

Women face socio-economic and healthcare challenges throughout their lives and are often vulnerable to multiple barriers, including cultural constraints, financial limitations, and health-related obstacles [47]. Developing countries are often more challenging, with population at risk in these areas are more likely to face greater risk due to poor living conditions, limited access to healthcare, and ongoing conflicts [48, 49]. Moreover, cultural rigidity and gender bias adds to this burden, resulting in the vulnerability of women to accessing healthcare services [50, 51]. Therefore, considering these factors, this study aimed to understand the health care access barriers among currently married women in Afghanistan. Our results show a significant and clear correlation between barriers to accessing healthcare and socio-economic and other risk factors, including asset ownership and women’s empowerment.

Results from our study show significant and positive association between women living in rural areas and barriers to health care accessibility. These findings corroborated earlier research, where greater risk was associated with healthcare accessibility among rural women [52]. The main reasons for these barriers are the limited access to healthcare facilities and socio-economic disadvantages experienced by rural women, in contrast to their urban counterparts. Additionally, factors such as limited transportation options and financial resources make it even more difficult for women in rural areas to access high-quality healthcare services [52, 53].

Socio-economic factors, such as literacy, partners education and wealth status are key to greater accessibility of health care services and their utilization [54]. Women with higher literacy rates, educated partners, and belonging to wealthier demographics tend to have better access to healthcare facilities and encounter fewer barriers, as indicated by our findings. Our study unequivocally showed that illiterate women from impoverished backgrounds face heightened risks of encountering obstacles in accessing healthcare, compared to their educated counterparts and those from higher income brackets. These results align with various previous studies underscoring the pivotal role of education and income levels in healthcare access, both in Afghanistan and other developing countries [28, 52–54].

Work opportunity provides women better excess to health care utilization and enhance their empowerment through the availability and accessibility of resources to utilize these services. Our findings are in line with earlier studies, suggesting that employment status is associated with a lower likelihood of facing barriers in accessing healthcare services, specifically, working women were found to have a lower likelihood of experiencing such barriers [55].

Research shows that empowerment factors like autonomy in decision-making, wealth status, and asset ownership are essential for accessing health care services [56, 57]. However, in Afghanistan, these factors are vital barriers, as women are vulnerable to limited decision-making power and lack of asset ownership, which can hinder their autonomy and impact their health and wellbeing. The results of our study indicate that women with greater decision-making power are less likely to face barriers in accessing healthcare, a finding supported by previous research [58, 59].

The study also found a significant association between asset ownership and barriers to accessing health care. Women with greater ownership are less likely to encounter barriers in Afghanistan. This can be attributed to the fact that higher income affords women more autonomy and influence in household decision-making regarding healthcare and other wellbeing issues [60].

### Limitation of the study

To our knowledge this study is a first of its kind in Afghanistan to analyze barriers to accessing healthcare services and linking them with key factors like asset ownership and women empowerment. However, despite this, the study has inherent limitations primarily stemming from the reliance on secondary data which is not the latest in our case. The term women empowerment is broad and encompasses more than just decision-making and overcoming obstacles. But due to the nature of our data source, we had to confine our analysis to these two aspects. Similarly, our exploration of asset ownership was restricted to land and household, even though it could have been examined in a more comprehensive manner. Additionally, the use of secondary data introduces the potential for reporting bias. We also acknowledge that our study did not account for areas of conflict, a critical control variable that would have enhanced our analysis. Lastly, the cross-sectional nature of the data poses challenges in establishing causality in our findings.

## Conclusion

Maternal health access is a key policy challenge in Afghanistan, given the barriers currently, married women face in the country. The above results reveal a significant proportion of women facing barriers related to approval, money, distance, and company by a family member while accessing the health services in the country. Similarly, women empowerment and asset ownership were also significantly associated with the barriers to accessing healthcare services apart from education of their husbands. This paper recommends a comprehensive policy intervention to address the challenges faced by women in accessing healthcare. This intervention should focus on enhancing women’s healthcare needs, providing economic incentives to empower them, and removing perceived barriers to accessibility through awareness-raising campaigns and incentives. Moreover, the provision of healthcare services at the grassroot level can be essential in ensuring that poor and socio-economically marginalized women utilize healthcare services, thereby promoting health equity and improving the utilization of health services in Afghanistan.

### Supplementary Information

**Supplementary Material 1.**

## Data Availability

The data that supports the findings of this study are available on request. The dataset used in the study is available in the public domain and can be accessed on a request from DHS at https://dhsprogram.com/Data/. Dataset and materials used in this study are available on request from the corresponding author mohammad.rahman@manipal.edu.

## Abbreviations

AFDHS: Afghanistan Demographic Health Survey
MDG: Millennium Development Goals
SDG: Sustainable Development Goals
