# Supplementary material for: Barriers to healthcare utilization among married women in Afghanistan: the role of asset ownership and women’s autonomy
Source: BMC Public Health. 2024 Feb 26;24:613. doi: 10.1186/s12889-024-18091-y (PMC10898116; doi:10.1186/s12889-024-18091-y)
Supplement: Supplementary file 1 — Supplementary Material 1. [file 12889_2024_18091_MOESM1_ESM.docx]

**Data**

The 2015 Afghanistan Demographic and Health Survey (2015 AFDHS) is the first DHS survey conducted in Afghanistan. The main objective of the 2015 AFDHS is to provide up-to-date information on fertility and childhood mortality levels; fertility preferences; awareness, approval, and use of family planning methods; maternal and child health; and knowledge and attitudes toward HIV/AIDS and other sexually transmitted infections (STIs). All other detailed information on sampling and survey is provided on the DHS website in detail.

**Sampling Weights; AFDHS** used a two-stage probability sample. A probability sample is defined as one in which the units are selected randomly with known and nonzero probabilities. A sampling frame is a complete list of all sampling units that entirely covers the target population. Sampling weights are adjustment factors applied to each case in tabulations to adjust for differences in probability of selection and interview between cases in a sample, due to either design or happenstance. Thus, individual weights were used to compute the final estimates for over all sample.

**Missing Data:** We used data cleaning and other means to prepare the dataset. All the unnecessary information were removed, and the final sample was adjusted based on the available information on the dependent and independent variables

**Construction of Key Variables**

**Questions used for Health care utilization barriers.**

The outcome variable was barriers to accessing health care. In AFDHS, all women were asked, “When you are sick and want to get medical advice or treatment, is each of the following a big problem or not? (1) Getting permission to go to the doctor? (2) Getting money needed for advice or treatment? (3) The distance to the health facility? (4) Not wanting to go alone?” The outcome variable was recategorized by recoding “yes” responses to the items “getting permission to go to the doctor,” “getting money for advice or treatment,” and “not wanting to go alone and distance to the health care facility” Those women who encountered all or some problems in these four areas were categorized as having barriers in accessing health care and those who did not encounter any problem in the four domains were categorized as having no barriers.

**Questions used for Women’s Empowerment Variable**

**Decision Making**

1. Decision on large household purchase
2. Decision on visits to family and relatives
3. Decision on what to do with money husband earned
4. Decision on respondent’s healthcare

All the four variables were grouped together to generate a composite score from 0 to 4. The composite score was then categorized into a binary variable with 0 meaning not deciding anything on its own, whereas 1 for 1 to four with any having any power to decide in anyone of the following in the study.

**Reasons for justifying beating**

1. Beating justified if wife goes out without telling husband
2. Beating justified if wife neglects
3. Beating justified if wife neglects the children the children
4. Beating justified if wife argues with husband
5. Beating justified if wife refuses to have sex Beating justified if wife burns the food

A composite score was developed based on women’s attitudes towards justification of wife beating based on the set of five questionnaires as mentioned above. Finally, the score was computed into a binary variable with 0 as those who did not justify whereas 1 for those justifying beating for any of the above reasons in the study.

**Household Ownership**

Household ownership was computed based on two questions asked to respondents about house and land ownership. These two variables then grouped together to generate a single variable with 0 for not owning any of these and 1 as ownership of any one from the two.

**Covariates**

**Wealth index**

The wealth index is a composite measure of a household's cumulative living standard. The wealth index is calculated using easy-to-collect data on a household's ownership of selected assets, such as televisions and bicycles; materials used for housing construction; and types of water access and sanitation facilities in the AFDHS.

**Place of Residence**: Place of residence classifies the sample population into rural and urban households in the data.

Other covariates include women’s age, education, ethnicity, region and so on.

**Figure SI: Diagrammatic representation of access to health care utilization and women empowerment**

**Access to Healthcare (Outcome Variable)**

- Permission to Go
- Getting Money
- Not Wanting to go Alone
- Barriers in distance to health facility

**Other Covariates**

- Age of Mother
- Residence
- Number of Living Children
- Respondent Education
- Husband Education
- Wealth Index

**Women Empowerment (Exposure Variable)**

- Household Asset Ownership
- Justifying Beating
- Household decision making

**Table TI: Description of Variables**

| **Variable** | **Definition** |
| --- | --- |
| **Dependent Variable** | |
| **Barriers to access healthcare** | Permission to go to the doctor (0,1)  Getting money for receiving treatment, (0,1)  Distance from health facility (0,1)  Not wanting to go alone. (0,1)  **For Bivariate**  (0 = no barriers at all, 1 = had faced one barrier, 2 = had faced more than one barrier)  **For Multivariate**  (0 = no barriers at all, (1 & 2 Any Barriers) |
| **Exposure Variables** | |
| **Decision Making** | Decision on large household purchase (0= No,1=Yes)  Decision on visits to family and relatives (0= No,1=Yes)  Decision on what to do with money husband earned (0= No,1=Yes)  Decision on respondent’s healthcare (0= No,1=Yes) |
| **Reason for Justifying Women** | Beating justified if wife goes out without telling husband (0= No,1=Yes)  Beating justified if wife neglects (0= No,1=Yes)  Beating justified if wife neglects the children the children (0= No,1=Yes)  Beating justified if wife argues with husband (0= No,1=Yes)  Beating justified if wife refuses to have sex Beating justified if wife burns the food (0= No,1=Yes) |
| **Household Ownership** | Respondent owning a House (0= No,1=Yes)  Respondent owning a Land (0= No,1=Yes) |
| **Other Covariates** | |
| Age in Groups | (15-19=1), (20-29=2), (30-39=2) and (40-49=3) |
| Residence | (Urban=1) (Rural=2) |
| Number of Living Children | No Children=1, Up to 2=2, Three to Four=3 and 5+=4 |
| Mothers and Fathers Education | No Education=1, Primary, Secondary and Higher |
| Mothers Working | Not Working=0 and working = 1 |
| Wealth Index | Poorest=0, Poorer=1, Middle=2, Richer=3 and Richest =5 |
